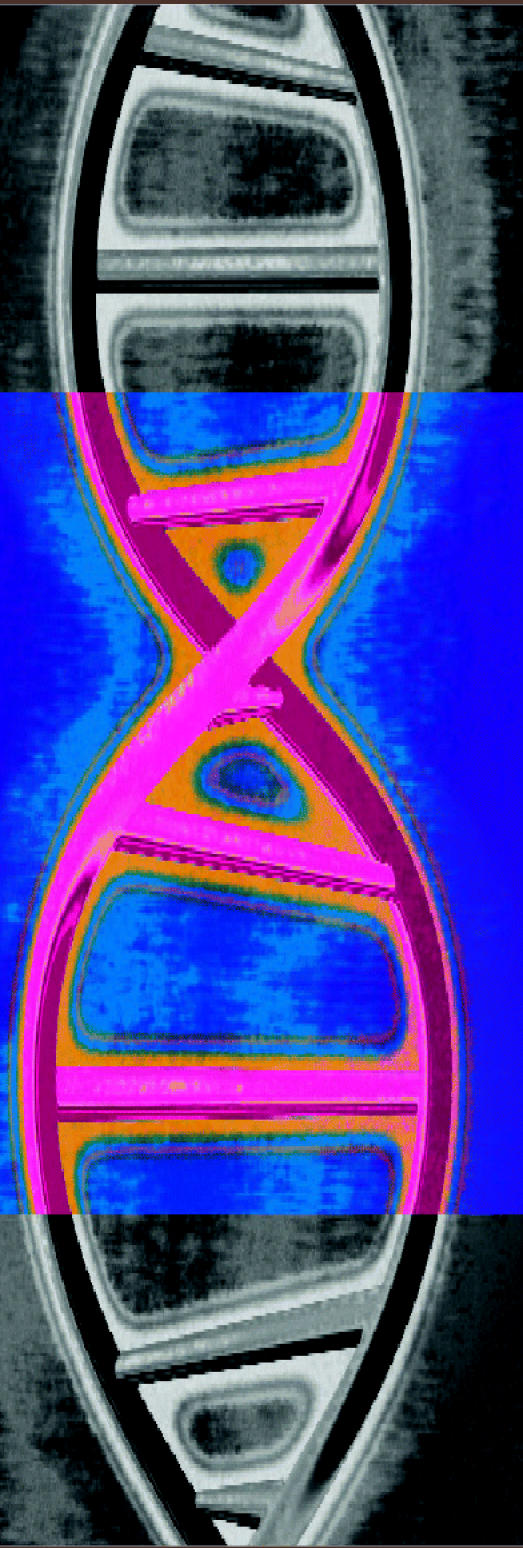# Epigenetics: The Science of Change

**DOI:** 10.1289/ehp.114-a160

**Published:** 2006-03

**Authors:** Bob Weinhold

For nearly a century after the term “epigenetics” first surfaced on the printed page, researchers, physicians, and others poked around in the dark crevices of the gene, trying to untangle the clues that suggested gene function could be altered by more than just changes in sequence. Today, a wide variety of illnesses, behaviors, and other health indicators already have some level of evidence linking them with epigenetic mechanisms, including cancers of almost all types, cognitive dysfunction, and respiratory, cardiovascular, reproductive, autoimmune, and neurobehavioral illnesses. Known or suspected drivers behind epigenetic processes include many agents, including heavy metals, pesticides, diesel exhaust, tobacco smoke, polycyclic aromatic hydrocarbons, hormones, radioactivity, viruses, bacteria, and basic nutrients.

In the past five years, and especially in the past year or two, several groundbreaking studies have focused fresh attention on epigenetics. Interest has been enhanced as it has become clear that understanding epigenetics and epigenomics—the genomewide distribution of epigenetic changes—will be essential in work related to many other topics requiring a thorough understanding of all aspects of genetics, such as stem cells, cloning, aging, synthetic biology, species conservation, evolution, and agriculture.

## Multiple Mechanisms

The word “epigenetic” literally means “in addition to changes in genetic sequence.” The term has evolved to include any process that alters gene activity without changing the DNA sequence, and leads to modifications that can be transmitted to daughter cells (although experiments show that some epigenetic changes can be reversed). There likely will continue to be debate over exactly what the term means and what it covers.

Many types of epigenetic processes have been identified—they include methylation, acetylation, phosphorylation, ubiquitylation, and sumolyation. Other epigenetic mechanisms and considerations are likely to surface as work proceeds. Epigenetic processes are natural and essential to many organism functions, but if they occur improperly, there can be major adverse health and behavioral effects.

Perhaps the best known epigenetic process, in part because it has been easiest to study with existing technology, is DNA methylation. This is the addition or removal of a methyl group (CH_3_), predominantly where cytosine bases occur consecutively. DNA methylation was first confirmed to occur in human cancer in 1983, and has since been observed in many other illnesses and health conditions.

Another significant epigenetic process is chromatin modification. Chromatin is the complex of proteins (histones) and DNA that is tightly bundled to fit into the nucleus. The complex can be modified by substances such as acetyl groups (the process called acetylation), enzymes, and some forms of RNA such as microRNAs and small interfering RNAs. This modification alters chromatin structure to influence gene expression. In general, tightly folded chromatin tends to be shut down, or not expressed, while more open chromatin is functional, or expressed.

One effect of such processes is imprinting. In genetics, imprinting describes the condition where one of the two alleles of a typical gene pair is silenced by an epigenetic process such as methylation or acetylation. This becomes a problem if the expressed allele is damaged or contains a variant that increases the organism’s vulnerability to microbes, toxic agents, or other harmful substances. Imprinting was first identified in 1910 in corn, and first confirmed in mammals in 1991.

Researchers have identified about 80 human genes that can be imprinted, although that number is subject to debate since the strength of the evidence varies. That approximate number isn’t likely to rise much in years to come, writes a team including Ian Morison, a senior research fellow in the Cancer Genetics Laboratory at New Zealand’s University of Otago, in the August 2005 *Trends in Genetics*. Others in the field disagree. Randy Jirtle, a professor of radiation oncology at Duke University Medical Center, and his colleagues estimated in the June 2005 issue of *Genome Research* that there could be about 600 imprinted genes in mice; in an October 2005 interview Jirtle said he’s anticipating a similar tally for humans, even though the known imprintable genes of mice and people have an overlap of only about 35%.

## Links to Disease

Among all the epigenetics research conducted so far, the most extensively studied disease is cancer, and the evidence linking epigenetic processes with cancer is becoming “extremely compelling,” says Peter Jones, director of the University of Southern California’s Norris Comprehensive Cancer Center. Halfway around the world, Toshikazu Ushijima is of the same mind. The chief of the Carcinogenesis Division of Japan’s National Cancer Center Research Institute says epigenetic mechanisms are one of the five most important considerations in the cancer field, and they account for one-third to one-half of known genetic alterations.

Many other health issues have drawn attention. Epigenetic immune system effects occur, and can be reversed, according to research published in the November–December 2005 issue of the *Journal of Proteome Research* by Nilamadhab Mishra, an assistant professor of rheumatology at the Wake Forest University School of Medicine, and his colleagues. The team says it’s the first to establish a specific link between aberrant histone modification and mechanisms underlying lupus-like symptoms in mice, and they confirmed that a drug in the research stage, trichostatin A, could reverse the modifications. The drug appears to reset the aberrant histone modification by correcting hypoacetylation at two histone sites.

Lupus has also been a focus of Bruce Richardson, chief of the Rheumatology Section at the Ann Arbor Veterans Affairs Medical Center and a professor at the University of Michigan Medical School. In studies published in the May–August 2004 issue of *International Reviews of Immunology* and the October 2003 issue of *Clinical Immunology*, he noted that pharmaceuticals such as the heart drug pro-cainamide and the antihypertensive agent hydralazine cause lupus in some people, and demonstrated that lupus-like disease in mice exposed to these drugs is linked with DNA methylation alterations and interruption of signaling pathways similar to those in people.

## Substantial Changes

Most epigenetic modification, by whatever mechanism, is believed to be erased with each new generation, during gameto-genesis and after fertilization. However, one of the more startling reports published in 2005 challenges this belief and suggests that epigenetic changes may endure in at least four subsequent generations of organisms.

Michael Skinner, a professor of molecular biosciences and director of the Center for Reproductive Biology at Washington State University, and his team described in the 3 June 2005 issue of *Science* how they briefly exposed pregnant rats to individual relatively high levels of the insecticide methoxychlor and the fungicide vinclozolin, and documented effects such as decreased sperm production and increased male infertility in the male pups. Digging for more information, they found altered DNA methylation of two genes. As they continued the experiment, they discovered the adverse effects lasted in about 90% of the males in all four subsequent generations they followed, with no additional pesticide exposures.

The findings are not known to have been reproduced. If they are reproducible, however, it could “provide a new paradigm for disease etiology and basic mechanisms in toxicology and evolution not previously appreciated,” says Skinner. He and his colleagues are conducting follow-up studies, assessing many other genes and looking at other effects such as breast and skin tumors, kidney degeneration, and blood defects.

Other studies have found that epigenetic effects occur not just in the womb, but over the full course of a human life span. Manel Esteller, director of the Cancer Epigenetics Laboratory at the Spanish National Cancer Center in Madrid, and his colleagues evaluated 40 pairs of identical twins, ranging in age from 3 to 74, and found a striking trend, described in the 26 July 2005 issue of *Proceedings of the National Academy of Sciences*. Younger twin pairs and those who shared similar lifestyles and spent more years together had very similar DNA methylation and histone acetylation patterns. But older twins, especially those who had different lifestyles and had spent fewer years of their lives together, had much different patterns in many different tissues, such as lymphocytes, epithelial mouth cells, intra-abdominal fat, and selected muscles.

As one example, the researchers found four times as many differentially expressed genes between a pair of 50-year-old twins compared to 3-year-old twins, and the 50-year-old twin with more DNA hypomethylation and histone hyperacetylation (the epigenetic changes usually associated with transcriptional activity) had the higher number of overexpressed genes. The degree of epigenetic change therefore was directly linked with the degree of change in genetic function.

Sometimes the effects of epigenetic mechanisms show up in living color. Changes in the pigmentation of mouse pup fur, ranging from yellow to brown, were directly tied to supplementation of the pregnant mother’s diet with vitamin B_12_, folic acid, choline, and betaine, according to studies by Jirtle and Robert Waterland published in August 2003 (issue 15) in *Molecular and Cellular Biology*. The color changes were directly linked to alterations in DNA methylation. In a study forthcoming in the April 2006 issue of *EHP*, Jirtle and his colleagues also induced these alterations through maternal ingestion of genistein, the major phytoestrogen in soy, at doses comparable to those a human might receive from a high-soy diet. The methylation changes furthermore appeared to protect the mouse offspring against obesity in adulthood, although there are hints that genistein may also cause health problems, via additive or synergistic effects on DNA methylation, when it interacts with other substances such as folic acid.

## Other Drivers of Change

Substances aren’t the only sources of epigenetic changes. The licking, grooming, and nursing methods that mother rats use with their pups can affect the long-term behavior of their offspring, and those results can be tied to changes in DNA methylation and histone acetylation at a glucocorticoid receptor gene promoter in the pup’s hippocampus. This finding was published in the August 2004 issue of *Nature Neuroscience* by Moshe Szyf, a professor in McGill University’s Department of Pharmacology and Therapeutics, and his colleagues. In the same study, the researchers found that the effects weren’t written in stone; giving the drug trichostatin A to older pups could help reverse the effects of poor maternal care received when they were younger. In the 6 June 2003 *Journal of Biological Chemistry* and the 23 November 2005 *Journal of Neuroscience*, Szyf and many of the same colleagues also demonstrated that giving the amino acid l-methionine to older pups could negate the benefits of high-quality maternal care received when they were younger.

Along with behavior, mental health may be affected by epigenetic changes, says Arturas Petronis, head of the Krembil Family Epigenetics Laboratory at the Centre for Addiction and Mental Health in Toronto. His lab is among the first in the world, and still one of only a few, to study links between epigenetics and psychiatry. He and his colleagues are conducting large-scale studies investigating links between schizophrenia and aberrant methylation, and he says understanding epigenetic mechanisms is one of the highest priorities in human disease biology research. “We really need some radical revision of key principles of the traditional genetic research program,” he says. “Epigenetics brings a new perspective on the old problem and new analytical tools that will help to test the epigenetic theory.” He suggests that more emphasis is needed on studying non-Mendelian processes in diseases such as schizophrenia, asthma, multiple sclerosis, and diabetes.

The past decade has also been productive in developing strong links between aberrant DNA methylation and aging, says Jean-Pierre Issa, a professor of medicine at The University of Texas M.D. Anderson Cancer Center. He presented information on aging and epigenetic effects at a November 2005 conference titled “Environmental Epigenomics, Imprinting, and Disease Susceptibility,” held in Durham, North Carolina, and sponsored in part by the NIEHS. Some of the strongest, decade-old evidence shows progressive increases in DNA methylation in aging colon tissues, and more recent evidence links hypermethylation with atherosclerosis. Altered, age-related methylation has also been found in tissues in the stomach, esophagus, liver, kidney, and bladder, as well as the tissue types studied by Esteller. Much of Issa’s current work focuses on the links between epigenetic processes, aging, the environment, and cancer, and possible ways to therapeutically reverse methylation linked with cancer.

## Current and Future Quandaries

The accumulated evidence indicates that many genes, diseases, and environmental substances are part of the epigenetics picture. However, the evidence is still far too thin to form a basis for any overarching theories about which substances and which target genes are most likely to mediate adverse effects of the environment on diseases, says Melanie Ehrlich, a biochemistry professor at the Tulane University School of Medicine and Tulane Cancer Center who has been conducting research on the topic for more than two decades.

That sense of uncertainty generally leaves epigenetics out of the regulatory picture. “It’s [too early] to actually use it at the moment,” says Julian Preston, acting associate director for health at the EPA’s National Health and Environmental Effects Research Laboratory. But Preston says the agency already relies more on its improving understanding of mechanistic processes, including epigenetics, and there is a clear effort within the EPA to expand genomics efforts both within the agency and with others with whom the agency works.

At the FDA, scientists are investigating many drugs that function through epigenetic mechanisms (although as spokes-woman Christine Parker notes, the agency bases its approvals on results of clinical trials, not consideration of the mechanism by which a drug works). One such drug, azacitidine, has been approved for use in the United States to treat myelodysplastic syndrome, a blood disease that can progress to leukemia. The drug turns on genes that had been shut off by methylation. The drug’s epigenetic function doesn’t make it a “miracle drug,” however. Trials indicate it benefits only 15% of those who take it, and a high percentage of people suffer serious side effects, including nausea (71%), anemia (70%), vomiting (54%), and fever (52%).

Ehrlich points out that azacitidine also has effects at the molecular level—such as inhibiting DNA replication and apoptosis—that may be part of its therapeutic benefits. The drug’s mixed results might also be explained in part by a study published in the October 2004 issue of *Cancer Cell* by Andrew Feinberg, director of the Johns Hopkins University Center for Epigenetics in Common Human Disease, and his colleagues. They found that each of two tested drugs, trichostatin A and 5-aza-2′-deoxycytidine (which is related to azacitidine), can turn on hundreds of genes while also turning off hundreds of others. If that finding holds in other studies, it suggests one key reason why it is so difficult to create a drug that doesn’t cause unintended side effects.

## Public and Private

Despite the potentially huge role that epigenetics may play in human disease, investment in this area of study remains tiny compared to that devoted to traditional genetics work. Several efforts to change that are under way.

In Europe, the Human Epigenome Project was officially launched in 2003 by the Wellcome Trust Sanger Institute, Epigenomics AG, and the Centre National de Génotypage. The group’s focus is on DNA methylation research tied to chromosomes 6, 13, 20, and 22. They may be joined soon by organizations in Germany and India, where scientists plan to work on chromosomes 21 and X, respectively, says Sanger senior investigator Stephan Beck.

But comprehensively studying all the epigenetic and epigenomic factors related to a multitude of diseases and health conditions will take much more work. “A [comprehensive] Human Epigenome Project is a lot more complicated than a Human Genome Project,” Jones says. “There’s only one genome, [but] an epigenome varies in each and every tissue.” The Human Genome Project was a worldwide effort that took more than a decade and billions of dollars to complete.

Jones and Robert Martienssen addressed some of the complexities of a comprehensive, worldwide Human Epigenome Project in the 15 December 2005 issue of *Cancer Research*. Reporting on a June 2005 workshop convened by the American Association for Cancer Research, they concluded that, despite all the looming difficulties, such a project is essential, and the technology is sufficiently advanced to begin.

“I think it’s going to happen a lot sooner than I thought just a year or so ago,” Jirtle says. A group of researchers has already started the footwork to launch a U.S. complement to the European Human Epigenome Project effort [see box, p. A165].

Other efforts are gaining ground. Another European group, the Epigenome Network of Excellence, took off in June 2004. This information exchange network includes members in the public and private sectors spread throughout ten Western European countries. Their objectives are to coordinate research, provide mentors, and encourage dialogue via their website. And in Asia, a conference held 7–10 November 2005 in Tokyo, “Genome-Wide Epigenetics 2005,” was dedicated in large part to facilitating a coordinated epigenomics research effort in Japan and possibly all of Asia, says Ushijima, one of the conference’s organizers.

In the United States, the National Cancer Institute and the National Human Genome Research Institute formally kicked off a major effort 13 December 2005 that will include epigenomic work. The pilot project of The Cancer Genome Atlas, funded by $50 million each from the two institutes, is designed to lay the groundwork for comprehensive study of genomic factors related to human cancer. The initial three-year effort is expected to focus on just two or three of the more than 200 cancers known to exist, but if it’s successful in developing methods and technologies, the number of cancers evaluated could then expand. If a high number of cancer genes are eventually scrutinized, the effort would be the equivalent of thousands of Human Genome Projects.

To help push the boundaries further, the NIEHS and the National Cancer Institute are in the midst of awarding grants totaling $3.75 million to study a wide range of epigenetic topics, such as identification of high-risk populations, dietary influences on cancer, and detailed study of numerous specific mechanisms linking environmental agents with epigenetic mechanisms and resulting disease. The dozen or so recipients are expected to launch their projects by fall 2006.

The NIEHS has also begun to integrate epigenomics projects into its research portfolio over the past five to six years. “It’s an emerging area that’s very important,” says Frederick Tyson, a program administrator in the NIEHS Division of Extramural Research and Training. And epigenetics is likely to be one of the half dozen or so most important considerations as NIEHS proceeds with its Environmental Genome Project, according to institute director David Schwartz.

The DNA Methylation Society, a professional group, has been growing slowly but steadily over the past decade, says founder and current vice president Ehrlich. As part of its efforts, the society launched a journal, *Epigenetics*, in January 2006 with the goal of covering a full spectrum of epigenetic considerations—medical, nutritional, psychological, behavioral—in any organism. Such groups are a valuable rallying point for this field, Jirtle says. He himself slowly worked his way into epigenetics from an initial cancer focus, and his segue is typical of many. “If you study epigenetics, you don’t have a home; we come from all different fields,” he says.

Interest in the private sector is also picking up. For instance, Epigenomics AG, with offices in Berlin and Seattle, is working on early detection and diagnosis of cancer and endometriosis (for which there is limited evidence of an epigenetic component), as well as development of products to predict effectiveness of drugs to treat these diseases. Founded in 1998, and now with about 150 employees, the company is focusing on DNA methylation mechanisms, and is working with companies such as Abbott Laboratories, Johnson & Johnson, Philip Morris, Roche Diagnostics, Pfizer, and AstraZeneca. CEO Oliver Schacht says the surging interest in this field is typified by the difference between the 2004 American Association for Cancer Research conference, which had half a dozen or so talks or posters on epigenetics, and the 2005 event, which had about 200.

## Tool Time

If epigenetic work is to continue breaking new ground, many observers say technology will need to continue advancing. Jones and Martienssen note in their paper that there must be additional improvements in high-throughput technologies, analytical techniques, computational capability, mechanistic studies, and bioinformatic strategies. They also say there is a need for basics such as standardized reagents and a consistent supply of antibodies for testing.

Preston agrees with many of these ideas, and says there is also a need to develop a comprehensive tally of all proteins in the cell and to get better protein modification information. He says universities are recognizing the demand for the talents needed to solve epigenomics problems, and are increasing their efforts to cover these topics in various ways, especially at the graduate school level.

Other groups are doing their part by creating tools to further the field. All the imprinted genes identified so far are tracked in complementary efforts by Morison’s and Jirtle’s groups and the Mammalian Genetics Unit of the U.K. Medical Research Council. The European managers of the DNA Methylation Database have assembled a compendium of known DNA methylations that, although not comprehensive, still provides a useful tool for researchers investigating the roughly 22,000 human genes.

Kunio Shiota, a professor of cellular biochemistry at the University of Tokyo and one of the co-organizers of the November 2005 Tokyo conference, says epigenetic advances will rely in part on a range of processes that are slowly becoming familiar to more researchers—massively parallel signature sequencing (MPSS), chromatin immunoprecipitation microarray analysis (ChIP-chip), DNA adenine methyltransferase identification (Dam-ID), protein binding microarrays (PBM), DNA immunoprecipitation microarray analysis (DIP-chip), and more. Someday, he says, these terms could become fully as familiar as MRI and EKG.

The rapidly growing acceptance of epigenetics, a century after it first surfaced, is a huge step forward, in Jirtle’s opinion. “We’ve done virtually nothing so far,” he says. “I’m biased, but the tip of the iceberg is genomics and single-nucleotide polymorphisms. The bottom of the iceberg is epigenetics.”

## Resources

**Professional Organizations and Projects**

- DNA Methylation Society (international) http://www.dnamethsoc.com/main.htm
- Epigenome Network of Excellence (Europe) http://www.epigenome-noe.net
- Human Epigenome Project (Europe) http://www.epigenome.org

**Journal**

Epigenetics

http://www.landesbioscience.com/journals/epigenetics/

**DNA Methylation Database**

http://www.methdb.de/front.html

**Imprinted Gene Databases**

- http://igc.otago.ac.nz/home.html
- http://www.geneimprint.com/databases/?c=clist
- http://www.mgu.har.mrc.ac.uk/research/imprinting/

## U.S. Human Epigenome Project

In December 2005 a group of 40 international scientists publicly proposed a U.S. Human Epigenome Project to complement a European project of the same name launched in 2003. Group member Andrew Feinberg, a geneticist at the Johns Hopkins University School of Medicine, says, “We’re hoping to see how this idea takes hold. There is this ocean of information that is largely unexplored.”

The goal of the U.S. project will be to comprehensively map methylation and histone modifications —the two main classes of epigenetic modifications—in a diverse set of normal tissues. These epigenomes would then serve as a reference for comparison with diseased tissues, revealing epigenetic causes of disease. Project organizers are now compiling a detailed proposal, with budget estimates and a timeline.

Although both the U.S. and European projects ultimately aim to map all genes, the U.S. effort will look at different tissue and cell types than the European effort, and will also look at model organisms like yeast and the fly. The two groups are already working closely together in planning their projects to avoid redundancies, and this cooperation will likely continue.

Understanding cancer would be one long-term goal for the U.S. project, but epigenetics—changes in gene expression heritable from cell to daughter cell without changes in DNA sequence—transcends any one disease. “It has profound implications in aging, neurological disorders, and child development,” says Peter Jones, another group member and director of the Norris Comprehensive Cancer Center at the University of Southern California. Jones and his colleagues argue that the importance of epigenetics in human disease, together with the maturing of technologies for mapping epigenetic changes, make a human epigenome project both critical and feasible.

Epigenetics, says cancer biologist Jean-Pierre Issa of The University of Texas M.D. Anderson Cancer Center, could prove more important than genetics for understanding environmental causes of disease. “Cancer, atherosclerosis, Alzheimer’s disease [are all] acquired diseases where the environment very likely plays an important role,” he points out. “And there’s much more potential for the epigenome to be affected … than the genome itself. It’s just more fluid and more easy to be the culprit.” **– Ken Garber**

## Figures and Tables

**Figure:**
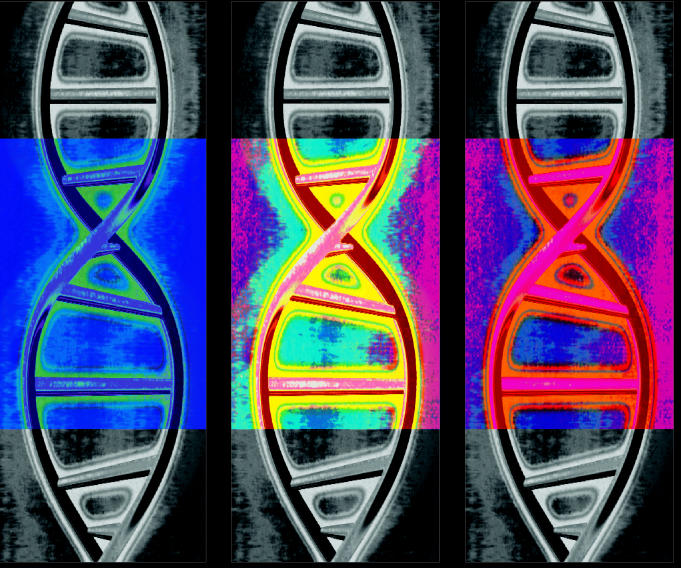


**Figure:**
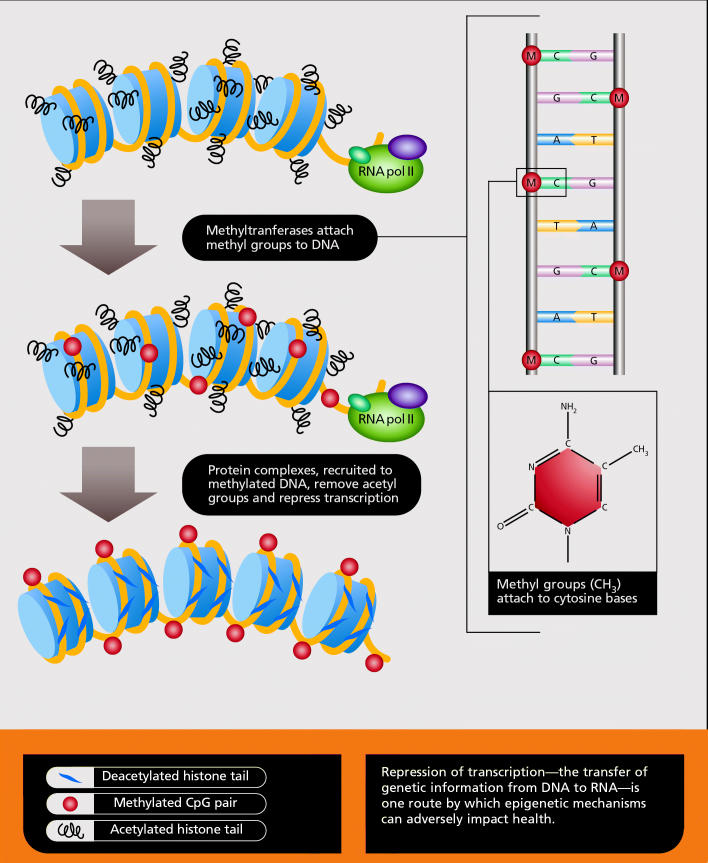
One Epigenetic Mechanism for Repressing Transcription

**Figure:**
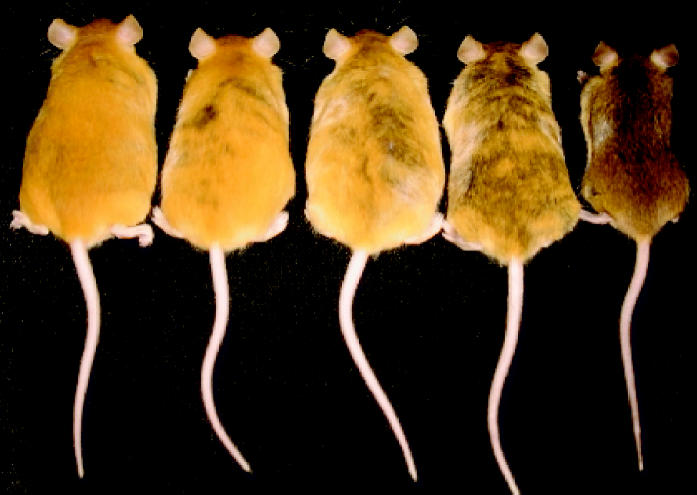
A pup of a different color. Supplementation of maternal diet with genistein and other compounds induced alterations in DNA methylation that were reflected in offspring coat color changes.

**Figure:**